# Supplementary material for: Stone decision engine accurately predicts stone removal and treatment complications for shock wave lithotripsy and laser ureterorenoscopy patients
Source: PLoS One. 2024 May 2;19(5):e0301812. doi: 10.1371/journal.pone.0301812 (PMC11065282; doi:10.1371/journal.pone.0301812)
Supplement: S1 File — (DOCX) [file pone.0301812.s001.docx]

**Supplementary Materials**

**Stone Decision Engine accurately predicts stone removal and treatment complications for extracorporeal shock wave lithotripsy and laser ureterorenoscopy patients**

**Authors:** Peter A Noble^1*^, Blake D. Hamilton^2^, Glenn Gerber^3^.

**Affiliations**

^1^Department of Microbiology, University of Alabama Birmingham, Birmingham, AL 35294.

^2^School of Medicine, University of Utah, Salt Lake City, USA 84132

^3^University of Chicago Medical Center, Chicago, IL 60637

*Correspondence to: Peter A Noble: panoble2017@gmail.com, Phone: 206-409-6664.

**Draft version date**

January 17, 2024

**Contents**

1. FIGURES S1 -- S4
2. TABLES S1 – S16
3. **FIGURES S1 – S4**

*
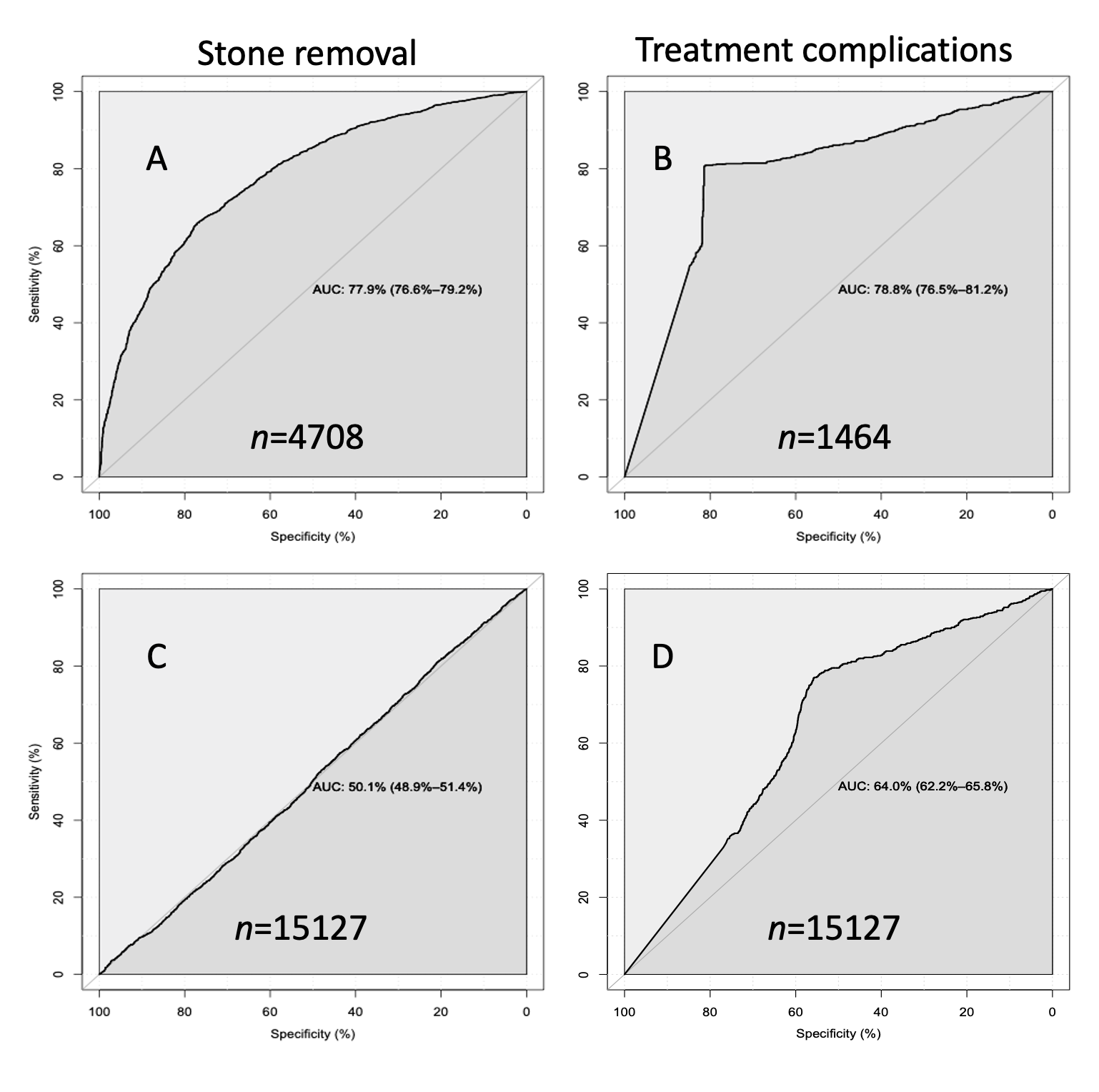
*

**Fig. S1. AUCs of SWL stone removal and treatment complications for ANN models built using balanced data sets. A, B; AUCs based on model tested with 70% training/30% testing and validation data sets; C, D; AUCs obtained by testing the models on the entire dataset.**


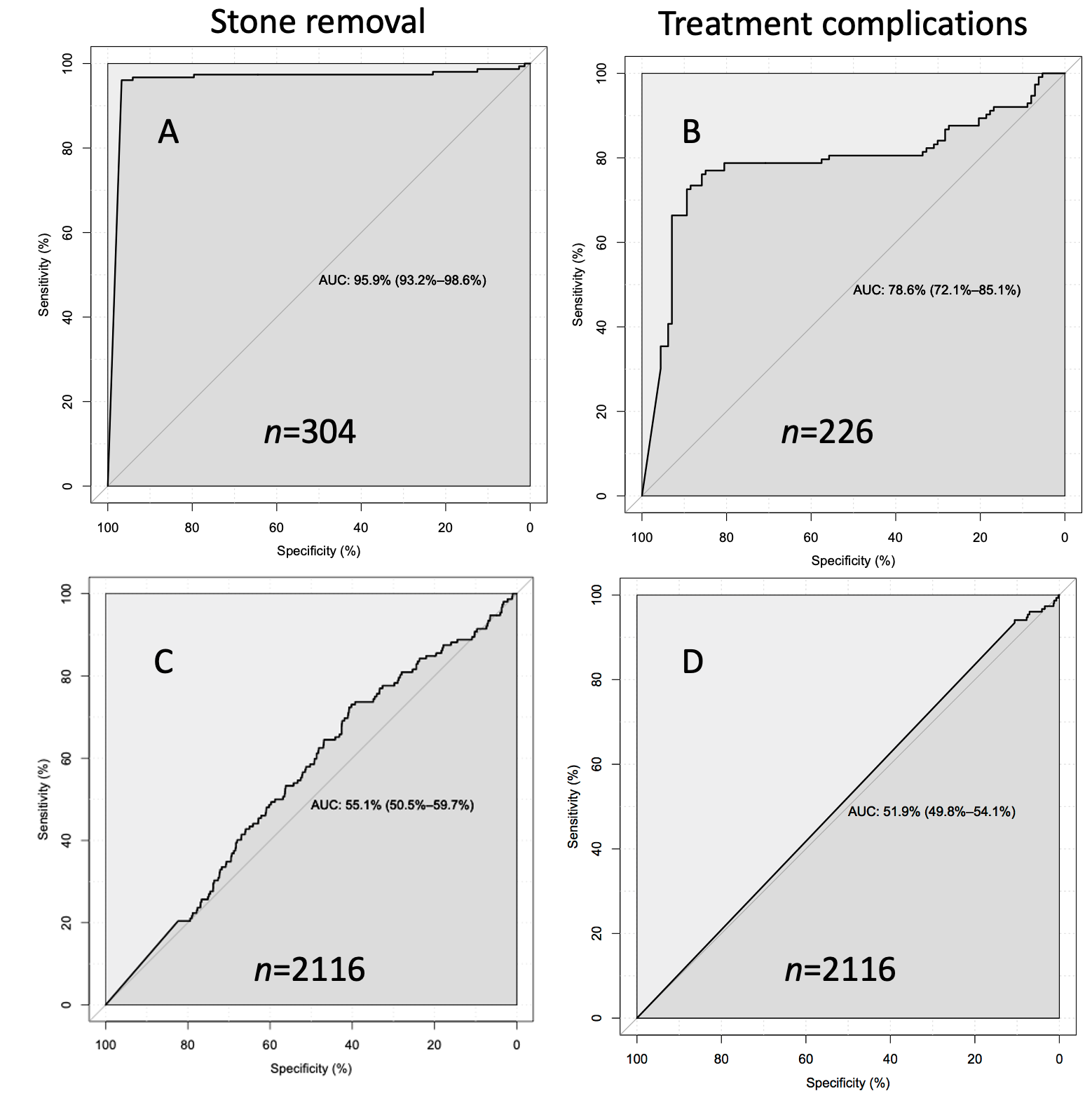


**Fig. S2. AUCs of URS stone removal and treatment complications for ANN models built using balanced data sets and tested with entire data sets. A, B; AUCs based on models tested with 70% training/30% test and validation data sets; C, D; AUCs obtained by testing the models on the entire dataset.**


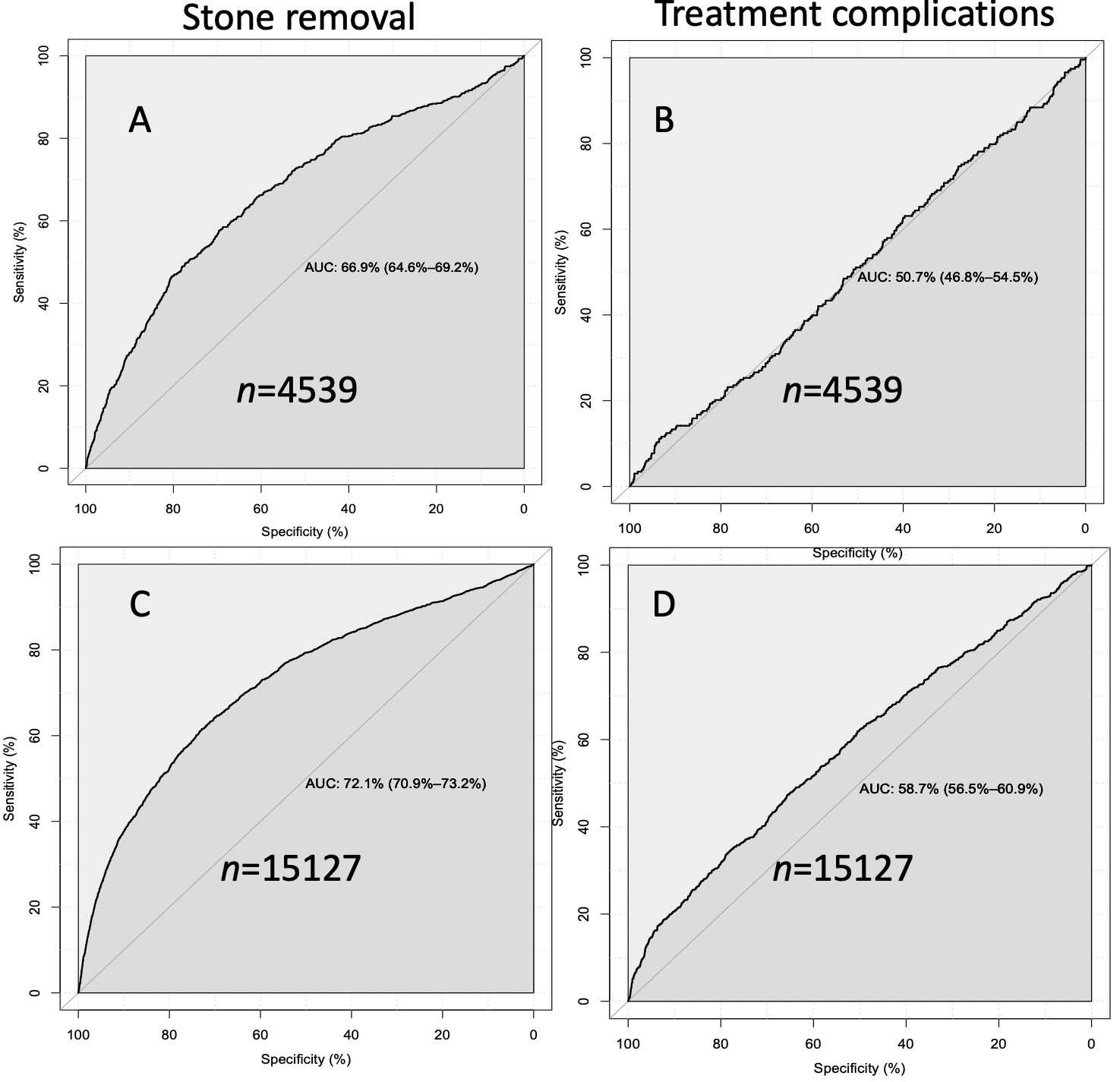


**Fig. S3. AUCs of SWL stone removal and treatment complications for ANN models built using SMOTED data sets and tested with validation (A, B) and the entire data set (C, D).**


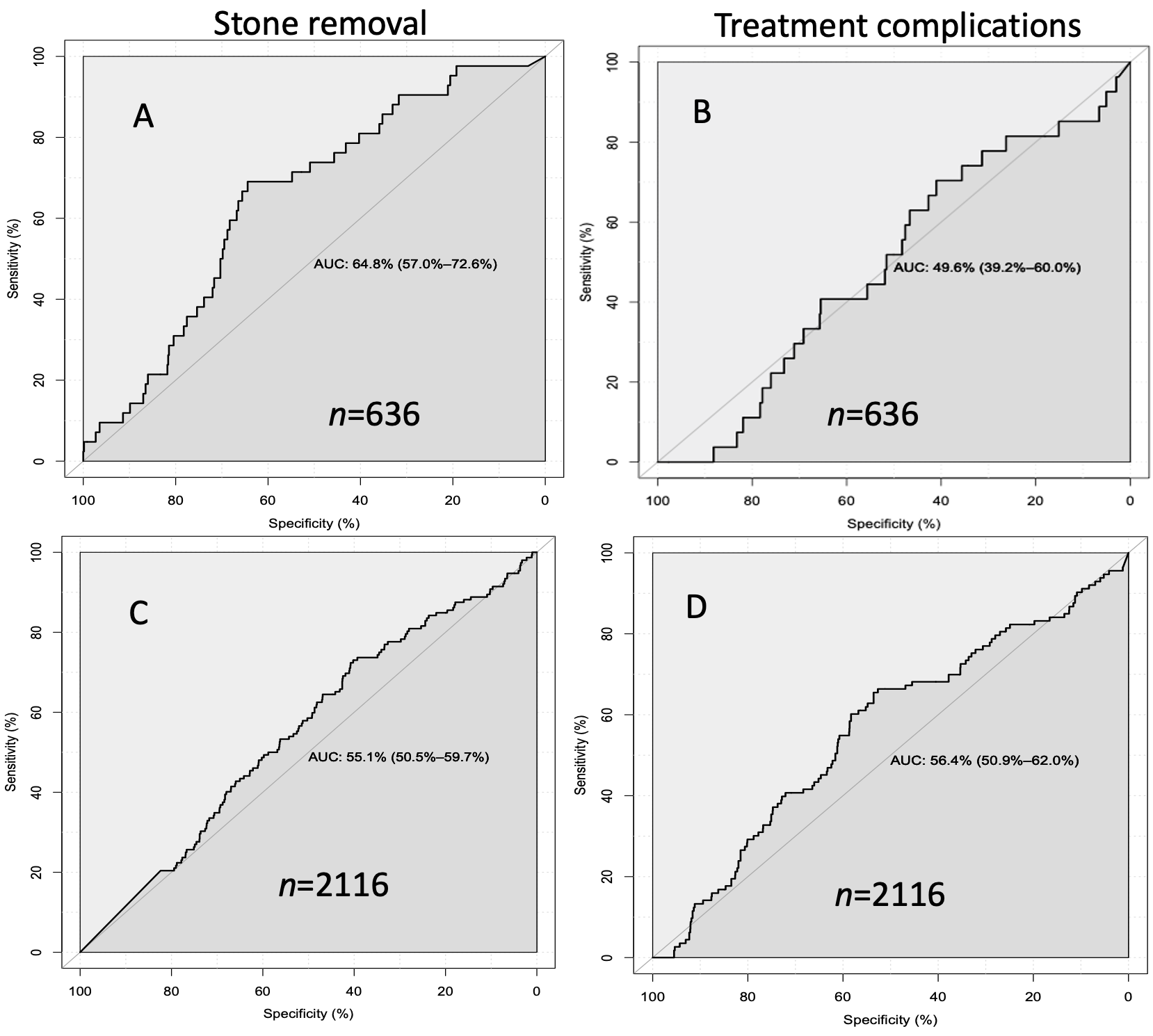


**Fig. S4. AUCs of URS stone removal and treatment complications for ANN models built using SMOTED data sets and tested with validation (A, B) and the entire data set (C, D).**

1. **TABLES S1 – S16**

**Table S1. Confusion matrix of SWL ‘Stone removal’ using balanced data set (*n*=4708 records).**

| **Actual (below) /Predictions (across)** | **0** | **1** | **Sum** |
| --- | --- | --- | --- |
| 0 | 38.7% (*n*=1824) | 11.2% (*n*=530) | 2354 |
| 1 | 15.0% (*n*=710) | 34.9% (*n*=1644) | 2354 |
|  |  |  | 73.7% (*n*=4708) |

0, stone removal success; 1, stone removal failure.

**Table S2. Confusion matrix of SWL ‘Stone removal’ model (Table S1) using the entire data set (*n*=15127 records).**

| **Actual (below) /Predictions (across)** | **0** | **1** | **Sum** |
| --- | --- | --- | --- |
| 0 | 17.6% (*n*=2663) | 66.8% (*n*=10110) | 12773 |
| 1 | 11.0% (*n*=1659) | 4.6% (*n*=694) | 2353 |
|  |  |  | 22.2% (*n*=15126) |

0, stone removal success; 1, stone removal failure.

**Table S3. Confusion matrix of SWL ‘Treatment complication’ model using balanced data set (*n*=1466 records).**

| **Actual (below) /Predictions (across)** | **0** | **1** | **Sum** |
| --- | --- | --- | --- |
| 0 | 40.5% (*n*=594) | 9.4% (*n*=138) | 732 |
| 1 | 9.6% (*n*=140) | 40.5% (*n*=594) | 734 |
|  |  |  | 81.0% (*n*=1466) |

0, No treatment complications; 1, Treatment complication

**Table S4. Confusion matrix of SWL ‘Treatment complications’ model (Table S3) using the entire data set (*n*=15127 records).**

| **Actual (below) /Predictions (across)** | **0** | **1** | **Sum** |
| --- | --- | --- | --- |
| 0 | 52.9% (*n*=8004) | 42.2% (*n*=6391) | 14395 |
| 1 | 1.1% (*n*=167) | 3.7% (*n*=564) | 731 |
|  |  |  | 56.6% (*n*=15126) |

0, No treatment complications; 1, Treatment complication

**Table S5. Confusion matrix of URS ‘Stone removal’ using a balanced data set (*n*=304 records).**

| **Actual (below) /Predictions (across)** | **0** | **1** | **Sum** |
| --- | --- | --- | --- |
| 0 | 46.4% (*n*=141) | 3.6% (*n*=11) | 152 |
| 1 | 3.6% (*n*=11) | 46.4% (*n*=141) | 152 |
|  |  |  | 92.8% (*n*=304) |

0, stone removal success; 1, stone removal failure.

**Table S6. Confusion matrix of URS ‘Stone removal’ model (Table S5) using the entire data set (*n*=2116 records).**

| **Actual (below) /Predictions (across)** | **0** | **1** | **Sum** |
| --- | --- | --- | --- |
| 0 | 9.2% (*n*=195) | 83.6% (*n*=1769) | 1964 |
| 1 | 0.4% (*n*=9) | 6.8% (*n*=143) | 152 |
|  |  |  | 16.0% (*n*=2116) |

0, stone removal success; 1, stone removal failure.

**Table S7. Confusion matrix of URS ‘Treatment complications’ using a balanced data set (*n*=226 records).**

| **Actual (below) /Predictions (across)** | **0** | **1** | **Sum** |
| --- | --- | --- | --- |
| 0 | 43.4% (*n*=98) | 6.6% (*n*=15) | 113 |
| 1 | 13.3% (*n*=30) | 36.7% (*n*=83) | 113 |
|  |  |  | 80.1% (*n*=226) |

0, No treatment complications; 1, Treatment complications.

**Table S8. Confusion matrix of URS ‘Treatment complications’ model (Table S7) using the entire data set (*n*=2116 records).**

| **Actual (below) /Predictions (across)** | **0** | **1** | **Sum** |
| --- | --- | --- | --- |
| 0 | 59.2% (*n*=1252) | 35.5% (*n*=751) | 2003 |
| 1 | 1.4% (*n*=30) | 3.9% (*n*=83) | 113 |
|  |  |  | 63.1% (*n*=2116) |

0, No treatment complications; 1, Treatment complications.

**Table S9. Confusion matrix of SMOTED SWL stone removal success model using validation data set (hold out; n=4539).**

| **Actual (below) /Predictions (across)** | **0** | **1** | **Sum** |
| --- | --- | --- | --- |
| 0 | 80% (*n=*3613) | 5% (*n=*220) | 3833 |
| 1 | 13%(*n=*571) | 3% (*n=*135) | 706 |
|  |  |  | 82.5% (*n*=4539) |

0, stone removal success; 1, stone removal failure.

**Table S10. Confusion matrix of SMOTED SWL stone removal success model using entire data set (n=15127).**

| **Actual (below) /Predictions (across)** | **0** | **1** | **Sum** |
| --- | --- | --- | --- |
| 0 | 81% (*n=*12185) | 4% (*n=*588) | 12773 |
| 1 | 12% (*n=*1796) | 4% (*n=*557) | 2353 |
|  |  |  | 84.2% (*n*=15126) |

0, stone removal success; 1, stone removal failure.

**Table S11. Confusion matrix of SMOTED SWL treatment complications model using validation data (hold out; n=4539).**

| Actual/Predictions | 0 | 1 |  |
| --- | --- | --- | --- |
| 0 | 94% (*n*=4260) | 1% (*n*=46) | 4306 |
| 1 | 5% (*n*=226) | 0% (*n*=7) | 233 |
|  |  |  | 94.0% (*n*=4539) |

0, No treatment complications; 1, Treatment complications.

**Table S12. Confusion matrix of SMOTED SWL treatment complications model using entire data set (n=15126).**

| **Actual (below) /Predictions (across)** | **0** | **1** | **Sum** |
| --- | --- | --- | --- |
| 0 | 94% (*n*=14257) | 1% (*n*=138) | 14395 |
| 1 | 5% (*n*=693) | 0% (*n*=38) | 731 |
|  |  | s | 94.4% (*n*=15126) |

0, No treatment complications; 1, Treatment complications.

**Table S13. Confusion matrix of SMOTED URS stone removal model using validation data set (hold out; n=636).**

| **Actual (below) /Predictions (across)** | **0** | **1** | **Sum** |
| --- | --- | --- | --- |
| 0 | 89% (*n*=564) | 5% (*n*=29) | 593 |
| 1 | 6% (*n*=38) | 1% (*n*=4) | 42 |
|  |  |  | 89.4% (*n*=635) |

0, stone removal success; 1, stone removal failure.

**Table S14. Confusion matrix of SMOTED URS stone removal model using entire data set (n=2116).**

| **Actual (below) /Predictions (across)** | **0** | **1** | **Sum** |
| --- | --- | --- | --- |
| 0 | 93% (*n*=1964) | 0% (*n*=0) | 1964 |
| 1 | 7% (*n*=152) | 0% (*n*=0) | 152 |
|  |  |  | 88.6% (*n*=2116) |

0, No treatment complications; 1, Treatment complications.

**Table S15. Confusion matrix of SMOTED URS treatment complications model using validation data set (hold out; n=636).**

| **Actual (below) /Predictions (across)** | **0** | **1** | **Sum** |
| --- | --- | --- | --- |
| 609 | 88% (*n*=557) | 8% (*n*=52) | 609 |
| 27 | 4% (*n*=23) | 1% (*n*=4) | 27 |
|  |  |  | 88.2% (*n*=636) |

0, No treatment complications; 1, Treatment complications.

**Table S16. Confusion matrix of SMOTED URS treatment complications model using entire data set (n=2116).**

| **Actual (below) /Predictions (across)** | **0** | **1** | **Sum** |
| --- | --- | --- | --- |
| 0 | 92% (*n*=1955) | 2% (*n*=48) | 2003 |
| 1 | 5% (*n*=108) | 0% (*n*=5) | 113 |
|  |  |  | 92.6% (*n*=2116) |

0, No treatment complications; 1, Treatment complications.
